# Hepatitis E Virus Entry

**DOI:** 10.3390/v11100883

**Published:** 2019-09-20

**Authors:** Xin Yin, Zongdi Feng

**Affiliations:** 1 Center for Vaccines and Immunity, The Research Institute at Nationwide Children’s Hospital, Columbus, OH 43205, USA; 2 Department of Pediatrics, The Ohio State University College of Medicine, Columbus, OH 43210, USA

**Keywords:** quasienveloped virus, receptor, lysosomal acid lipase, NPC1

## Abstract

Hepatitis E virus (HEV) infection is a major cause of acute hepatitis worldwide. It is transmitted enterically but replicates in the liver. Recent studies indicate that HEV exists in two forms: naked, nonenveloped virions that are shed into feces to mediate inter-host transmission, and membrane-cloaked, quasienveloped virions that circulate in the bloodstream to mediate virus spread within a host. Both virion types are infectious, but differ in the way they infect cells. Elucidating the entry mechanism for both virion types is essential to understand HEV biology and pathogenesis, and is relevant to the development of treatments and preventions for HEV. This review summarizes the current understanding of the cell entry mechanism for these two HEV virion types.

## 1. Introduction

Hepatitis E virus (HEV) infection is a common cause of acute hepatitis worldwide [1]. Each year about 20 million individuals are infected with HEV, resulting in ~44,000 deaths. In recent years, increased cases have been reported in developed countries. In addition to humans, HEV also infects a wide range of animal species [2]. At least six different HEV genotypes have been shown to be able to infect humans. Most cases in developed countries are caused by zoonotic transmission of genotype 3 HEV through consumption of contaminated pork and game meat. In addition, genotype 3 HEV infections frequently cause persistent infections in individuals with a weakened immune system resulting in an increased risk for accelerated liver cirrhosis. HEV infection-related extrahepatic manifestations including various neurologic problems have also been described [2]. Despite these public health concerns, direct antivirals are not available for HEV. Ribavirin, a broad spectrum nucleoside analog, has been used in treating patients with chronic hepatitis E [3,4], but resistance mutations have been described [5,6]. There is an HEV vaccine available in China that offers long-term protections against clinically symptomatic acute hepatitis caused by both genotype 1 and 4 HEV infections [7,8]. This vaccine is currently under evaluation in a phase 1 clinical trial in the United States (https://clinicaltrials.gov/ct2/show/NCT03827395) and in a phase 4 clinical trial in Bangladesh (https://clinicaltrials.gov/ct2/show/NCT02189603).

Since its discovery in the 1980s, HEV has been considered as nonenveloped [9]. While this is true for virions in the bile and feces, it is now recognized that virions circulating in the bloodstream exist in a membrane associated, “quasienveloped” form (eHEV) [10,11]. eHEV particles are infectious, but they do not have classic envelope proteins thus infect cells via an unusual mechanism [12,13]. Elucidating the entry mechanisms for both virion types is critical for understanding the unique life cycle of HEV and its pathogenesis. Knowledge obtained from studying HEV entry may also aid in the development of antiviral drugs, which is crucial for patients with chronic hepatitis E experiencing ribavirin failure and in severe liver disease in pregnant women from developing countries. This review will begin with a discussion about the structural differences between the two HEV virion types, then discuss the current understanding regarding the entry mechanism for each virion type. 

## 2. Two Types of Virions Naturally Exist during an HEV Infection

**Figure 1:**
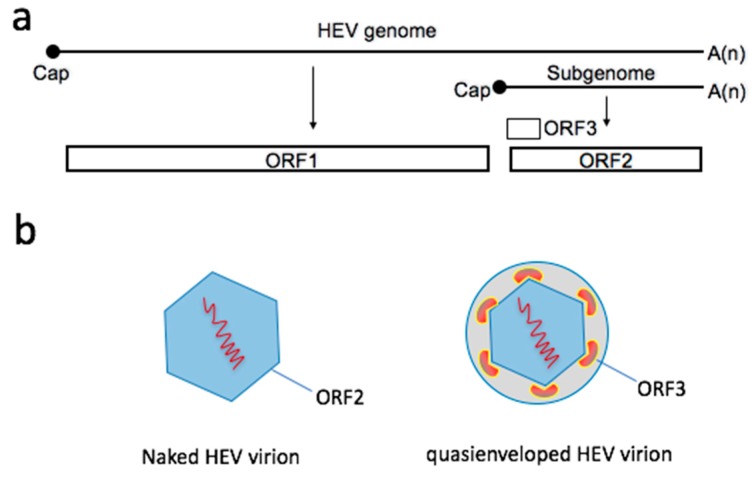
The hepatitis E virus (HEV) genome, its encoded proteins (**a**), and two types of virions (**b**).

HEV was discovered in 1983 by Dr. Mikhail Balayan, a Russian virologist who intentionally infected himself by ingesting pooled stool samples collected from HEV-infected Russian soldiers deployed in Afghanistan [9]. He subsequently developed acute hepatitis. By examining his fecal material using electron microcopy (EM), Dr. Balayan obtained the first morphologic evidence of HEV virions, which appeared as nonenveloped icosahedral particles, 27–30 nm in diameter, with a “spiky” surface [9]. Due to its similar structural appearance to the calicivirus, HEV was initially misclassified within the *Caliciviridae* family. In 1990, HEV was molecularly cloned and its full genome was sequenced [14]. The significant sequence divergence of HEV from other known families of viruses led to its reclassification into a new family, *Hepeviridae*. The 7.2 kb single-stranded positive sense HEV RNA genome was shown to be polyadenylated and contain three open reading frames (ORFs) (Figure 1a). The 5’ two-thirds of the genome encodes ORF1, a large multi-domain polyprotein that is involved in the genomic replication. The rest of the genome encodes ORF2 and ORF3, both of which are translated from a subgenomic RNA generated during the virus replication cycle. ORF2 is the only capsid protein of HEV. The ORF3 protein is unique to HEV and plays a role in virion egress [15]. A new ORF, ORF4, was recently discovered within the coding region of ORF1, but appears to be restricted only to genotype 1 HEV [16].

The ORF2 capsid protein of HEV contains 660 amino acids (a.a) that are divided into 3 structural domains: a shell (S) domain, a middle (M) domain, and a protruding (P) domain comprised of a.a. 129–319, 320–455, and 456–606, respectively [17]. Each virion consists of 90 copies of an ORF2 dimer, forming a *T* = 3 icosahedron. Recombinant ORF2 proteins expressed in the bacterial and insect cells can self-assemble into virus-like particles (VLPs), of which the crystal structures have been determined [17,18,19,20]. p239, a *T* = 1 VLP composed of a.a. 368–606 and produced in *E. coli*, is the main component of the vaccine Hecolin® currently marked in China. According to the 3D structure, the S domains form the base of the icosahedron, and the dimeric P domains form the “spikes” extending from the virion surface that are believed to be responsible for receptor binding and a main target for neutralizing antibodies [21]. 

The enteric route of transmission, EM evidence of naked virions in the feces, and the lack of coding capacity for envelope proteins all suggest that HEV is a nonenveloped virus. However, recent studies show that the virus released from infected cells and circulating in the blood adopts a membrane associated, “quasienveloped” form, named “eHEV” [10,11,22] (Figure 1b). These particles band at a much lower buoyant density (~1.10 g/cm^3^) in isopycnic gradient centrifugation than the traditional, nonenveloped virions (~1.25 g/cm^3^) [13], and are insensitive to neutralizing anti-capsid antibodies in standard neutralization assays [10]. Under EM, these particles look like enveloped viruses, with HEV capsids encased in limiting host-derived membranes [11]. Unlike naked HEV particles, the eHEV particles contain both ORF2 and ORF3 proteins. However, both of them are hidden within the host-derived membranes and can only be detected by specific antibodies upon disruption of the membrane by detergent [10]. eHEV is the only form detected in blood [23], suggesting that it mediates HEV spread within the host.

The biogenesis of eHEV has been reviewed elsewhere [24,25]. The prevalent model involves viral hijacking of the cellular endosomal sorting complex required for transport (ESCRT) machinery, producing an exosome-like vesicle containing the viral capsid and ORF3 protein. Usurping ESCRT components is a common mechanism for the budding of classic enveloped viruses [26]. The HEV ORF3 protein plays a key role in this process. A proline-serine-alanine-proline (PSAP) late domain motif located at the C-terminus of ORF3 specifically interacts with the cellular TSG101 (tumor susceptibility gene-1) protein, an ESCRT-I protein, which then recruits ESCRT-II and –III complexes to promote the budding of the HEV capsid into multivesicular bodies (MVB) [27]. Subsequent fusion of MVB with the plasma membrane leads to the release of single membrane-encased eHEV particles. Consistent with the biogenesis pathway for exosomes, eHEV membranes contain tetraspannins such as CD63, CD81, and CD9 [11,28]. Certain host proteins are likely copackaged during eHEV biogenesis, but their identity and roles in the HEV life cycle remain largely unexplored. A recent study used a quantitative proteomic approach to identify proteins associated with quasienveloped hepatitis A virus (eHAV) [29]. It has revealed that the eHAV particles are highly enriched for components of the endolysomal system such as CD9, DPP4 (dipeptidylpeptidase 4, CD26), ALIX, and EPCAM (epithelial cell-adhesion molecule), but lack LC-3 (an autophagosomal protein)-related peptides, consistent with an endosomal exosome-like origin of eHAV. This approach may be also useful for identifying eHEV-associated proteins.

The nonenveloped HEV particles found in the feces likely originated from eHEV released through the bile canaliculi, but their membrane are stripped by the detergent action of bile [22]. The lack of lipid membrane renders the nonenveloped virions much more stable in the environment to facilitate transmission to new hosts.

The production of two virion types is integral to HEV biology. Thus, it is important to understand how each virion type plays a role in the infection process. The available experimental evidence suggests that they enter and infect target cells through distinct pathways.

## 3. Cell Entry of Naked HEV

The naked, nonenveloped HEV particles are present in the bile and feces of infected patients or experimentally infected monkeys [23,30,31]. These particles are stable in the environment and ideal for transmission to new hosts. Although HEV is primarily transmitted enterically, how it penetrates the gut barrier to reach the bloodstream remains enigmatic. No compelling evidence exists that HEV replicates in the human gut, although it is possible that HEV infects only a rare cell type in the gut, as is the case for norovirus [32]. Regardless of the origin of the first cell to be infected, whether a hepatocyte or a yet-to-be-identified cell type in the gut, naked HEV is necessary for establishing the first round of infection.

Despite being highly hepatotropic in vivo, under in vitro conditions, HEV is able to infect a range of cell types other than hepatocytes, including A549 (human lung epithelial cells), Caco-2 (human colon epithelial cells), human neuronal-derived cells, and human placenta cells [33,34,35,36,37,38,39]. This widened cell tropism in vitro is not unique to HEV, as hepatitis A virus (HAV), which is known to only infect hepatocytes, can also infect many nonhepatic cell types in vitro [40]. It should be noted that cancer cell lines often bear genetic abnormalities and altered phenotypes as compared to their corresponding primary cells in vivo, thus results obtained from such cells must always be interpreted with caution. 

Study of the HEV life cycle has been hampered by the lack of efficient cell culture systems for HEV [41]. The particle to focus-forming unit (FFU) ratio for HEV ranges from 600 to 15,000, prohibiting the use of single particle imaging techniques to track the HEV entry process. Most published studies have used fluorescently labeled VLPs to study the early steps of cell entry including initial cell attachment and internalization. The recent development of HEV infection systems has provided the first opportunity to study the entire HEV life cycle in detail [38,39]. Below we summarize the current knowledge regarding the entry process of naked HEV particles. 

### 3.1. Cell Attachment

The receptor for HEV is unknown. However, a number of host factors have been shown to be involved in cell attachment and/or entry of naked HEV. These factors are summarized below.

#### 3.1.1. HSGPs (Heparan Sulfate Proteoglycans)

HSGPs are glycans present on the cell surface that are involved in cell attachment of many nonenveloped and enveloped viruses. HSGPs, particularly syndecans, have been shown to play a role in the binding of HEV VLPs to human hepatoma cells [42]. Treatment of cells with heparinase reduced VLP binding and HEV infection. However, HSGPs are not essential for cell attachment and infection by eHEV particles [13].

#### 3.1.2. GRP78 (Glucose-Regulated Protein 78)

GRP78, also known as BiP (binding immunoglobulin protein), is a molecular chaperone in the ER. However, presence of GRP78 on the cell surface has also been described and implicated in the attachment and entry of both enveloped and nonenveloped viruses [43,44,45,46]. GRP78 binds to p239 VLPs in both co-immunoprecipitation and a cell model [47].

#### 3.1.3. ASGPR (Asialoglycoprotein Receptor)

ASGPR is a protein receptor present on the basolateral membrane of hepatocytes that binds glycoproteins that lack sialic acid modifications. A direct interaction has been shown between the ectodomain of both ASGR1 and ASGR2 and HEV ORF2 by coimmunoprecipitation, pull-down, and ELISA [48]. Ectopic expression of ASGRP in HeLa cells increased HEV binding, whereas depletion of ASGPR in PLC/PRF/5 cells lowered HEV binding but not virion release. Both anti-ASGPR antibody and purified ASGPR ectodomain also reduced HEV binding to PLC/PRF/5 cells.

#### 3.1.4. ATP5B (ATP Synthase Subunit 5β)

ATP synthase is largely a mitochondrial protein, but a small fraction is expressed on the cell surface and is implicated in other viral infections [49]. ATP5B was identified as a binding partner on the p239 VLP using a pull down and mass spectrometric approach [50]. The role of ATP5B in HEV entry was validated using antibody and siRNA mediated approaches, and infectious HEV from the stool of a hepatitis E patient. 

#### 3.1.5. ITGA3 (Integrin Alpha 3)

Integrin α3 was recently identified as an entry factor for HEV in PLC/PRF/5 cells [51]. HEV permissive and nonpermissive subclones of PLC/PRF/5 have been identified, and compared to each other by microarray. Overexpression of integrin α3 in nonpermissive cells rendered cells permissive for HEV, while knocking out integrin α3 in permissive cells abrogated permissiveness. For unknown reasons, none of these subclones supported infection by eHEV particles. 

Although these results are encouraging, independent studies will be needed to validate the role of these factors in HEV and eHEV cell attachment and entry in the context of infection, preferably in primary human hepatocytes.

### 3.2. Internalization and Intracellular Trafficking

Using GFP- and firefly luciferase-tagged VLPs and naked HEV particles, Kapur et al. showed that HEV is internalized by cells in a clathin-, dynamin-dependent pathway [52]. An independent study using FITC-labeled VLPs subsequently confirms these results, and additionally shows that HEV-like particles initially traffic to Rab5-positive compartments, then to acidic lysosomal compartments where they are degraded [53]. The same study has also identified membrane cholesterol, the PI3K pathway, and actin as important factors for HEV internalization and infection, but low pH is not required. More recently, using naked HEV purified from cell lysates, we have shown that HEV infectivity is dependent on clathrin and dynamin 2, consistent with others. However, we found that the infectivity of naked HEV is not affected by inhibiting Rab5 and Rab7, or by lysosomotropic agents such as bafilomycin and ammonium chloride [13]. These results suggest that the uncoating of naked HEV particles occurs before reaching the Rab5+ compartment, and the co-localization of capsids to Rab5 observed by others likely represent empty capsids that remain in the endocytic pathway after genome release.

### 3.3. Uncoating

As a cytoplasmically replicating RNA virus, HEV has to deliver its genome across the endosomal membrane to access the cytoplasm for translation and replication. How this process takes place remains poorly understood. Studies with other nonenveloped RNA viruses have demonstrated that upon receptor binding the virions undergo structural rearrangements to cause the externalization of hydrophobic peptides [54]. These hydrophobic peptides subsequently insert into the endosomal membrane, creating a channel/pore through which the genome traversed the endosomal membrane to enter the cytoplasm. Viral capsids remaining in the endosome are ultimately degraded in the lysosomes. Since naked HEV particles do not colocalize with Rab5 and Rab7, HEV capsids may undergo substantial conformational changes during the uncoating process. 

## 4. Entry of Quasienveloped HEV

The presence of a quasienvelope raises several questions about the entry mechanism for eHEV. How does eHEV bind to cells and how is its cell tropism determined? Does eHEV entry involve membrane fusion? And does eHEV have a different uncoating mechanism from naked HEV? While there are no definitive answers for these questions, available evidence suggests that the eHEV quasienvelope is degraded by lysosomal enzymes prior to uncoating. If this model holds true, it would present a novel entry mechanism for viruses. 

### 4.1. Cell Attachment

Since quasi-enveloped HEV particles do not have viral proteins on the surface of their envelope, they must use different attachment factors and/or cellular receptors to initiate the viral entry. As with exosomes, the eHEV membrane has phosphatidylserine, which may bind to its receptor such as TIM-1 on the target cells [55,56]. Our previous study found that the cellular uptake of the eHEV virions is less efficient than naked HEV due to inefficient cell attachment, indicating that the undetermined molecules on the eHEV surface mediate the cell attachment with slower kinetics [13]. Unlike the naked HEV virion, the attachment of eHEV to the hepatocytes does not require HSPGs [13].

The less specific cell binding by eHEV may provide an explanation for the detection of HEV beyond the liver [57]. In addition, the exosome-like nature could facilitate its penetration of immunologically privileged sites such as the central nervous system. Since HEV infection has been associated with various types of extrahepatic manifestations [12], a better understanding of the tropism and replicative capacity of eHEV in relevant cells/tissues will help differentiate between virus-mediated and immune-mediated effects in these conditions and shed light on HEV pathogenesis.

### 4.2. Internalization and Intracellular Trafficking

Similar to naked HEV particles, eHEV enters hepatocytes mainly through the clathrin- and dynamin-dependent pathway [12,52]. Following the endocytic uptake, eHEV particles are transported sequentially into Rab5+ and Rab7+ endosomal compartments, and eventually reach the lysosome, where the uncoating is thought to take place [13,53]. Treatment of cells with lysosomotropic agents such as bafilomycin A1 and NH4Cl dramatically reduces eHEV infectivity, indicating that the endosomal acidification is required for eHEV entry. However, the low pH itself is not sufficient for eHEV cell entry, since extracellular exposure to low pH did not alter the density or infectivity of eHEV [13].

The study of eHAV has provided additional insights into the intracellular trafficking of quasienveloped viruses [58]. Confocal imaging experiments using fluorescently labelled eHAV particles suggests that intact eHAV is transported to the lysosome, but similarly labelled exosomes produced by uninfected cells do not colocalize with lysosomal markers. It is speculated that the eHAV membrane may contain a signal that drives its continued trafficking towards the lysosome [58]. Whether eHEV uses a similar mechanism for trafficking to the lysosome is unknown, but would be an interesting area for future investigation.

### 4.3. Uncoating

The presence of a quasienvelope would require the HEV capsid to penetrate two layers of membranes in order to deliver the viral genome into the cytoplasm. One possible way to achieve this is through fusion of the viral membrane with the cellular membrane. However, membrane fusion would result in the intact capsid, rather than the genome, entering the cytoplasm, likely a dead end for HEV. A second, more likely scenario is by removing the quasienvelope, so that the capsid will be exposed and available for binding to a receptor. 

**Figure 2:**
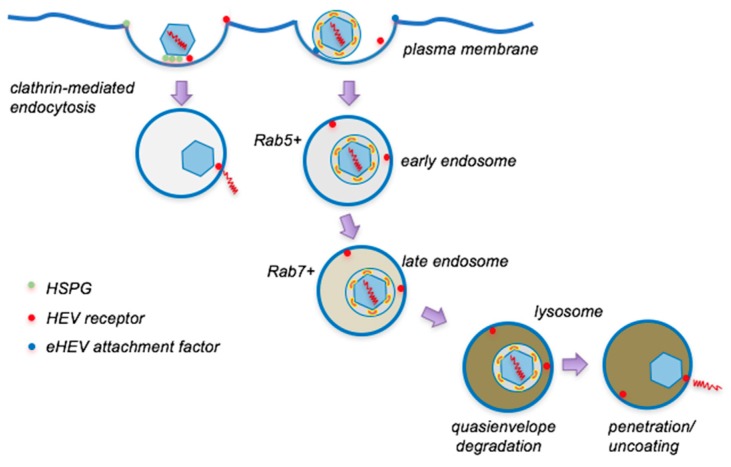
Model for cellular entry of naked and quasi-enveloped HEV virions. Naked HEV virions initially bind nonspecifically to HSPGs, followed by a specific interaction with a putative cell receptor. Receptor binding triggers virion internalization via clathrin-mediated endocytosis and virions subsequently uncoat in a Rab5− early endocytic compartment. Quasienveloped HEV virions attach to cells less efficiently than naked virions, but are similarly internalized via clathrin-mediated endocytosis. Virions are routed through early (Rab5+) and late (Rab7+) endocytic compartments and ultimately reach the lysosome. The quasi-envelope becomes slowly degraded by lysosomal enzymes, allowing the exposure of the capsid which subsequently penetrates the endosomal membrane to release its genome into the cytoplasm.

Lipid membrane degradation in lysosomes is a complex process [59]. A critical step for lipid membrane degradation is the Niemann-Pick disease type C1 (NPC1)-mediated extraction of cholesterol from lipids. Depletion of NPC1 reduced eHEV infection by 50% without significantly affecting HEV infectivity [13]. Moreover, cells pretreated with a specific inhibitor of lysosomal acid lipase (LAL), an enzyme essential in lipid metabolism because it hydrolyzes cholesteryl esters and triglycerides in lysosomes [60], displayed a dose-dependent reduction in eHEV infectivity, while no reduction was observed for non-enveloped HEV infectivity [13]. These results provide evidence that the eHEV membrane is degraded in the endolysosomes, rather than fusing with the host membrane (Figure 2). A similar requirement of NPC1 and LAL has also been described for eHAV entry [58].

Once eHEV loses its membrane, the capsid would be free to interact with its receptor on the endosomal membrane. At the simplest level, the capsid binds to the same receptor for naked HEV and uncoat via the same mechanism. Many cell surface proteins cycle between the plasma membrane and endosomes, serving as attractive candidates if this is the case. Alternatively, a different receptor may be used for eHEV capsids, and it is not uncommon for viruses to switch receptors during the entry process [61,62].

An unexplored question is the role of ORF3 in eHEV entry. ORF3 has been shown to interact with ORF2 [63]. Thus, the presence of ORF3 may interfere with receptor binding. In addition, ORF3 possesses an ion channel activity that is required for HEV egress [64]. It will be interesting to know whether this activity also plays a role in eHEV entry.

## 5. Summary

The presence of two distinct virion types has challenged our view about the fundamental biology and pathogenesis of HEV. The ability of HEV to infect its target cells both in the presence and absence of a viral membrane is a paradigm shift in virology and an exciting area for future investigations. The available evidence suggests that naked and quasienveloped HEV particles use different mechanisms for cell entry, and that entry of eHEV requires lysosomal degradation of the viral membrane. Identifying the cellular receptor for both virion types will be key to elucidating their entry mechanisms. Since eHEV is the only form detected in the bloodstream, understanding how eHEV spreads has the potential for identifying targets for intervention. With the recent development of more efficient culture systems for HEV, it is anticipated that our understanding of HEV entry will be improved within the next few years.
